# Single-Domain Antibodies as Therapeutic and Imaging Agents for the Treatment of CNS Diseases

**DOI:** 10.3390/antib8020027

**Published:** 2019-04-05

**Authors:** Kasandra Bélanger, Umar Iqbal, Jamshid Tanha, Roger MacKenzie, Maria Moreno, Danica Stanimirovic

**Affiliations:** 1 Human Health Therapeutics Research Centre, National Research Council Canada, Ottawa, ON K1A 0R6, Canada; Umar.Iqbal@nrc-cnrc.gc.ca (U.I.); Jamshid.Tanha@nrc-cnrc.gc.ca (J.T.); Colin.MacKenzie@nrc-cnrc.gc.ca (R.M.); Maria.Moreno@nrc-cnrc.gc.ca (M.M.); Danica.Stanimirovic@nrc-cnrc.gc.ca (D.S.); 2 Department of Biochemistry, Microbiology and Immunology, University of Ottawa, Ottawa, ON K1H 8M5, Canada

**Keywords:** single-domain antibodies, neurodegenerative diseases, brain imaging, blood–brain barrier, delivery

## Abstract

Antibodies have become one of the most successful therapeutics for a number of oncology and inflammatory diseases. So far, central nervous system (CNS) indications have missed out on the antibody revolution, while they remain ‘hidden’ behind several hard to breach barriers. Among the various antibody modalities, single-domain antibodies (sdAbs) may hold the ‘key’ to unlocking the access of antibody therapies to CNS diseases. The unique structural features of sdAbs make them the smallest monomeric antibody fragments suitable for molecular targeting. These features are of particular importance when developing antibodies as modular building blocks for engineering CNS-targeting therapeutics and imaging agents. In this review, we first introduce the characteristic properties of sdAbs compared to traditional antibodies. We then present recent advances in the development of sdAbs as potential therapeutics across brain barriers, including their use for the delivery of biologics across the blood–brain and blood–cerebrospinal fluid (CSF) barriers, treatment of neurodegenerative diseases and molecular imaging of brain targets.

## 1. Introduction to sdAbs

### 1.1. Structure and Characteristics

**Figure 1:**
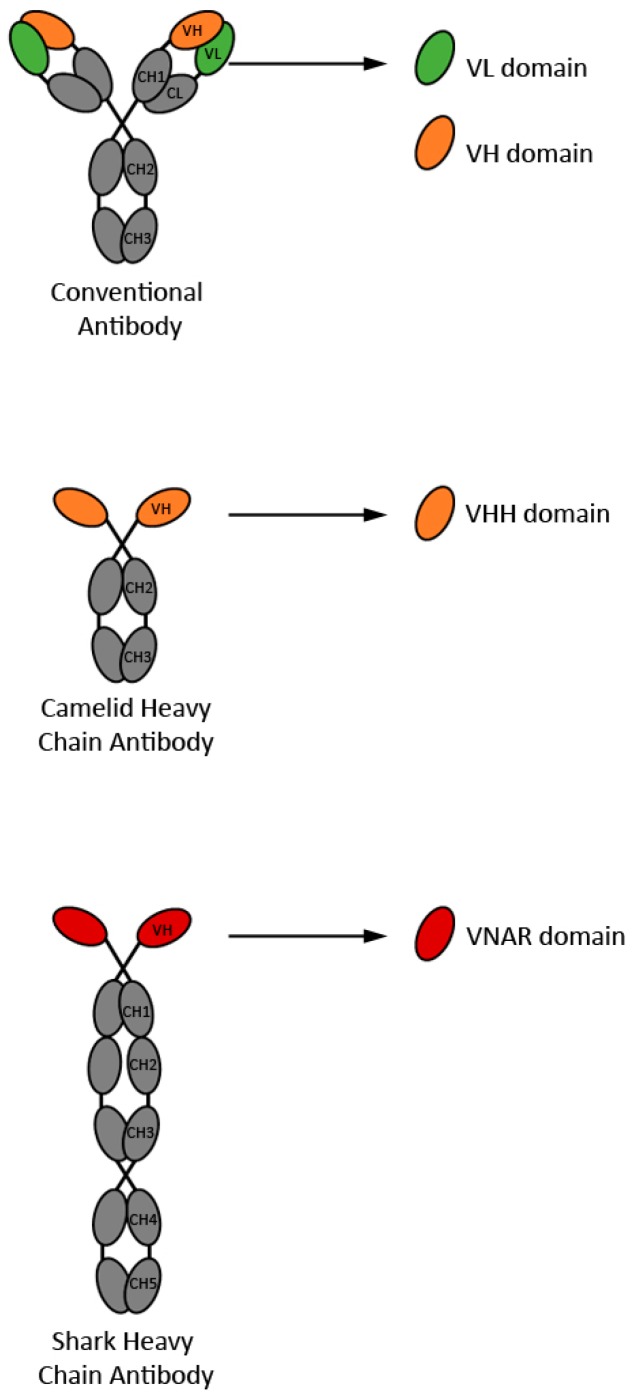
Schematic representation of the four types of sdAbs described in the current review. Antibody constant domains are in grey, whereas antibody variable domains from which sdAbs are derived are in color.

The concept of single-domain antibodies (sdAbs) originated in the 90’s, with the proof-of-concept experiments demonstrating sdAbs as *bone fide* antigen binding fragments [1], and the discovery of camelid [2] and shark [3] heavy chain-only antibodies (HCAbs). Single-domain antibodies can be derived from the antigen binding variable domains of homodimeric, light-chain lacking immunoglobulins, such as camelid HCAbs [4] and shark immunoglobulin new antigen receptors (IgNARs) [5], variable light chain (V_L_) or variable heavy chain (V_H_) domains of tetrameric—typically human—conventional immunoglobulins [6] (Figure 1). The variable domains of camelid HCAbs and shark IgNARs are referred to as V_H_Hs (or nanobodies) and V_NAR_s, respectively. While V_H_Hs and V_NAR_s are almost without exception non-aggregating and highly soluble, the opposite is true for V_H_s and V_L_s. However, various strategies have been developed to successfully obtain aggregation resistant and soluble human V_H_s and V_L_s ([7,8] and references therein) including transgenic mice technology [9,10]. Human V_H_ and V_L_ domains are of interest because of their human nature, a property that presumably makes them less immunogenic in humans compared to camelid V_H_Hs or nurse shark V_NAR_s.

The desirable biophysical, biochemical, and structural properties of sdAbs, particularly those from natural repertoires are generally well known, and have been described in several reviews [4,5,6,11,12]. Despite their significantly smaller combining site, consisting of only three complementarity-determining regions (CDRs) or hypervariable loops—as opposed to six for conventional antibodies such as monoclonal antibodies (mAbs)—sdAbs demonstrate comparable antigen binding affinities. Interrelated properties such as small size (12–15 kDa vs. 150 kDa for mAbs); strict monomericity; high solubility, including at therapeutic doses; aggregation resistance; chemical, physical and protease stability; efficient folding /refolding; good recombinant expression, notably in economic microbial expression systems such as yeast and *E. coli*; excellent shelf life; excellent manufacturability; and low cost of production make sdAbs an attractive alternative to other antibody formats such as mAbs, Fabs (fragments antigen binding), and scFvs (single chain variable fragments) as therapeutic and diagnostic agents. Resistance to aggregation is particularly noteworthy as it significantly reduces the risk of immunogenicity. Furthermore, their small size and frequently extended CDR3 make sdAbs the antibody of choice when targeting recessed epitopes of proteins such as enzymes’ active sites or receptors’ cavities. Longer CDR3s also increase the combining site’s surface area, and to a significant degree compensate for the absence of V_L_ CDRs. In addition, their fast blood clearance and effective tissue penetration, attributed to their small size, make sdAbs ideal imaging agents, e.g., against tumors. In this respect, the high stability and folding properties of sdAbs provide flexibility for labeling reactions with optimal outcomes. Modularity is another hallmark of sdAbs, and becomes a key property when engineering sdAb-based multimeric and multispecific constructs as CNS diagnostics and therapeutics (see Section 1.3).

### 1.2. Single-Domain Antibody Libraries and Selection

Single-domain antibodies have been typically isolated from display libraries mostly phage-displayed, although other display platforms also exist, such as yeast and ribosome display [13]. While V_H_Hs and V_NAR_s have been obtained from all types of libraries including immune, non-immune, semi-synthetic/synthetic libraries [4,14,15], human V_H_s and V_L_s have been most commonly obtained from synthetic libraries [6]. Unlike immune V_NAR_s and V_H_Hs that have high affinities as a result of in vivo somatic hypermutation, sdAbs obtained from non-immune or synthetic/semi-synthetic sdAb libraries are of low affinity, and often require further mutation for improved affinity and function. However, more recently, human V_H_s have also been isolated from immune V_H_ display libraries derived from HCAb-producing transgenic mice that are immunized with a target antigen of interest [9,10]. While these V_H_s display high solubility, stability and affinity of immune V_H_Hs, they are advantageously expected to be less immunogenic.

Constructing natural repertoire sdAb libraries is well established and relatively straightforward [4,15,16]. Human V_H_ and V_L_ synthetic libraries are typically built on a single scaffold with demonstrated good biophysical properties, such as high thermostability, solubility, and expression ([6,8] and references therein, [17,18]). Library diversity generation entails introducing random or specific amino acids at all or selected positions in the three CDRs. Owing to their small size and single-domain nature, in contrast to more complex, multidomain scFvs and Fabs, sdAbs lend themselves to a facile and straightforward library construction, and are not associated with V_H_/V_L_ mispairing phenomenon that occurs during the construction of scFv and Fab libraries, which adversely affects the library quality.

In its simplest and most commonly practiced format, the selection, or panning, the process for the isolation of sdAbs from phage-displayed libraries, involves selecting for a single property—affinity for the target antigen. Most commonly, this involves exposure of a library to an antigen immobilized on a microtitre plate, washing away unbound phage, and eluting the bound phage molecules, which are amplified for another round of panning. Depending on the type of library, between two and four rounds of panning are typically sufficient to obtain around half a dozen sdAbs with affinity for the target antigen. With human sdAb libraries, affinity selection may be coupled with selection for stability for a more efficient isolation of aggregation-resistant binders [6].

One of the great advantages of antibody library display technologies over hybridoma technology for the isolation of mAbs is the capability to drive, in some measure, the selection process towards isolating antibodies with specific properties. For example, by panning in the presence of proteases it has been possible to isolate sdAbs with enhanced protease resistance [19]. In the context of this review, it is especially noteworthy that sdAbs that transmigrate across an in vitro human blood–brain barrier (BBB) model have been isolated [20].

### 1.3. Modular Building of Multispecific Molecules

Their small size and monomeric nature make sdAbs ideal building blocks for the construction of multivalent and multispecific therapeutic and imaging molecules of improved function and potency (compared to monomeric versions) with good development capacity and manufacturability [11,21,22]. For example, bivalent or bispecific sdAbs have been generated by linking two identical or two different sdAbs using a short spacer sequence [23,24,25,26,27,28,29,30,31,32]. Successful generation of trivalent bispecific and tetravalent bispecific sdAbs—where sdAb moieties are linked through short linker sequences—have also been reported [28,29,33,34,35,36]. Monospecific pentavalent sdAbs have been constructed by fusing sdAbs to the N- or C-terminus of the verotoxin 1B (VT1B) subunit [37]. Similarly, fusing different sdAbs to the N- and C-terminus of VT1B has yielded bispecific decavalent molecules [38]. Bivalent monospecific or tetravalent bispecific sdAbs can also be made by fusing sdAbs to an antibody Fc fragment [8,39,40,41,42,43]; this has the added advantage of greatly extending the serum half-life of sdAbs [44] and imparting effector functions such as antibody-dependent cellular cytotoxicity (ADCC) [42] or complement-dependent cytotoxicity (CDC) [45]. Single-domain antibodies should also be ideal molecules for constructing bivalent and bispecific antibodies incorporating a heterodimeric Fc region [46]. More complex constructs such IgG-sdAb fusions have also been reported [47]. For therapeutic applications, sdAbs have been linked to enzymes or toxins, either by cloning or by chemical conjugation [48,49,50,51,52,53].

### 1.4. Developing sdAbs as CNS Diagnostics or Therapeutics

Treating CNS disorders remains one of the greatest challenges in modern medicine. Although several promising therapeutics are developed every year, their failure to reach brain target prevents their advancement to the clinic. This is mainly due to the presence of the BBB acting as a gatekeeper to maintain brain homeostasis and protect neurological capabilities [54]. The BBB is composed of specialized endothelial cells sealed together by tight junctions to form a physical barrier lining the brain blood vessels. These cells differ from endothelial cells lining peripheral vessels by their lack of fenestrations and limited pinocytic activity thereby restricting transcellular transport. The brain endothelial layer is surrounded by pericytes and astrocyte end-feet, which are essential for maintaining the integrity of the BBB. In addition, several efflux transporters are present at the BBB and function to remove unwanted molecules from the brain. Although this restrictive physiology is necessary to prevent undesirable blood-borne material from penetrating the brain, it also limits the effective delivery of CNS therapeutics. Therefore, agents designed for use as CNS diagnostics or therapeutics must be delivered to sites of action via administration routes that circumvent the BBB, such as intrathecal/intraventricular, intracerebral administration, or combined with delivery technologies that increase their penetration across the BBB upon systemic administration.

The brain neuropil is packed with interacting cells, including neurons, neuronal processes, and various types of glial cells. The brain extracellular space (ECS), filled with brain extracellular fluid, is tight and very convoluted—modeling studies estimate its width between 35 and 60 nm [55]. Any compound administered directly into the neuropil will diffuse through the ECS to distances inversely proportional to the size of the molecule. Monoclonal antibodies exhibit limited diffusion in the brain ECS due to their large size and interactions with the extracellular matrix (ECM). Single-domain antibodies have a distinct advantage as intracerebrally administered reagents/therapeutics, achieving diffusion across longer distances from the site of injection [55]. In addition, the lack of a Fc fragment reduces their interactions with the ECM and brain efflux via an FcRn-mediated reverse transcytosis. A recent study on the brain biodistribution of antibodies via perivascular transport after intrathecal infusion in rodents [56] demonstrated both deeper brain penetration and broader brain exposure of a smaller V_H_H fragment compared to a full mAb. This study demonstrated that sdAbs are advantageous as a CNS therapeutic antibody modality developed for intracerebral (local) or intrathecal administration. This is particularly relevant for brain diseases originating from or confined to a specific brain area, such as Parkinson’s disease.

However, the majority of CNS diseases can be considered ‘whole-brain’ diseases, even when they initially affect more localized brain regions. The brain’s vascular network is particularly dense, and thus each brain capillary supplies only few neurons, the diffusion distance of compounds, including antibodies, delivered across the BBB to their neuronal targets is only ~25 µm. Transvascular (cross-BBB) brain delivery would therefore achieve a more global brain distribution of antibodies, regardless of their size, since these diffusion distances are readily achievable even by mAbs. Systemic delivery of therapeutic antibodies targeting the CNS could be improved using ‘carrier’ molecules selected or engineered for the ability to traverse the BBB.

In the following sections, sdAbs that have been developed as delivery agents across the BBB, as treatments against the most common neurodegenerative diseases and as neuroimaging tools are descried and summarized in Table 1.

## 2. Single-Domain Antibodies as Delivery Agents across the BBB

The most investigated method to deliver macromolecules into the brain is via receptor-mediated transcytosis (RMT) [57]. This process is crucial for the proper delivery of macromolecules essential for brain function such as vitamins, proteins and nutrients. They use naturally occurring transport systems to shuttle between the blood and the brain. Such systems can potentially be ‘hi-jacked’ to facilitate the delivery of therapeutics into brain.

**Figure 2:**
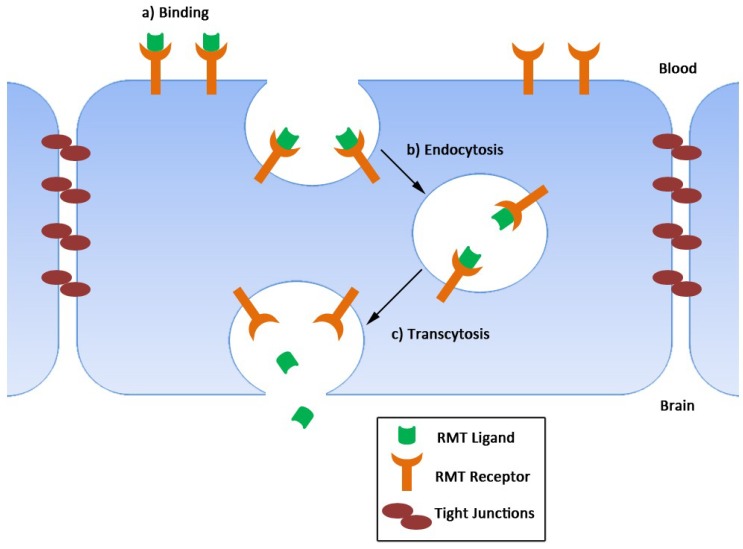
Representation of the receptor-mediated transcytosis (RMT) process. (**a**) Initially, an RMT ligand binds to a specific RMT receptor on the luminal cell membrane, which (**b**) leads to the internalization of both receptor and ligand in intracellular vesicles via endocytosis. (**c**) These vesicles then travel within the cell cytoplasm to reach the abluminal membrane where fusion of endosomes with the cell membrane releases the vesicular cargo inside the brain.

The process of RMT is initiated by ligand binding to its receptor expressed at the luminal face of endothelial cells to trigger internalization of the receptor-ligand complex into endosomal vesicles (Figure 2). These vesicles then travel inside the cytoplasm of the cell via a complex vesicular sorting pathway to finally fuse with the abluminal surface of endothelial cells and deliver their cargo in the brain parenchyma. The receptor is then recycled at the luminal cell surface. Currently, the main RMT receptors that have been studied are the transferrin receptor (TfR) and insulin receptor (IR) [57]. Ligands against these receptors, including different antibody formats, have been used as carriers to deliver their therapeutic cargo inside the brain [7,58,59,60,61,62,63,64,65]. Single-domain antibodies present numerous advantages over conventional antibodies as potential transvascular brain delivery vectors including small size, low non-specific interactions with tissues expressing high levels of Fc receptors (e.g., liver, spleen), remarkable stability against harsh conditions and low immunogenicity (see Section 1). In the case of IR, although there are examples of mAbs and peptides specific for this receptor, no IR-specific sdAbs have been described to date. The only example of a sdAb targeting TfR is a V_NAR_, termed TBX4, which was obtained from a synthetic library following a combination of in vitro and in vivo phage display techniques [66]. When fused to an immunoglobulin Fc backbone, this antibody was enriched in the brain parenchyma of mice following vein tail injection. Furthermore, bispecific variants of this antibody fused to a CD20 targeting agent were able to reach aberrant B cells in the brain and induced cell toxicity. The use of this V_NAR_ for the delivery of a variety of biologics to the brain is currently under investigation.

Although there is now growing evidence that the use of the aforementioned receptors for therapeutic delivery into the brain is a promising avenue, recent studies suggest that these may not be ideal RMT targets [67,68,69]. Major drawbacks associated with these receptors include their ubiquitous expression in numerous peripheral organs and their involvement in essential physiological functions, which may raise significant safety concerns. Therefore, the chase for alternative RMT receptors with more optimal BBB crossing properties continues.

FC5 and FC44, two camelid V_H_Hs of a non-immune phage-displayed library, were isolated by phenotypic panning for their ability to interact and internalize into the human brain cerebromicrovascular endothelial cells (HBECs) [20]. The two antibodies were found to transmigrate in an in vitro rat BBB model, and to accumulate in the brain at high levels following tail vein injection in rodents [20,70]. The investigation of their mechanism of action led to the finding that it was RMT-mediated [71], and in the case of FC5, the receptor was later identified as transmembrane domain protein 30A (TMEM-30A) [72]. Engineered fusions of FC5 to the human IgG1 Fc in a monovalent or bivalent format showed increased migration across the BBB in vitro, and achieved a significantly higher brain exposure in vivo compared to the control antibody-Fc [73]. FC5-Fc constructs were also detected in brain vessels and in the brain parenchyma in rat brain sections. Furthermore, the conjugation of FC5 antibodies with impermeable analgesic peptides, dalargin or neuropeptide Y, induced an important analgesic effect in a thermal hyperalgesia model whereas the systemic administration of the neuropeptides alone had no suppressive effect. Although all antibody formats were able to reduce hyperalgesia, the bivalent and monovalent Fc fusions showed a pronounced increase in the response at equal dose compared to the V_H_H suggesting that improving serum pharmacokinetics plays a determining role in the pharmacological potency of FC5. In addition to peptides, the CNS delivery of a monoclonal antibody antagonist of metabotropic glutamate receptor 1 (mGluR1) was successfully achieved using the BBB-crossing V_H_H FC5 [74]. Following intravenous injection in a rat model of persistent inflammatory pain, the BBB-mGluR1 bispecific antibody co-localized with thalamic neurons involved in mGluR1-mediated pain processing, and subsequently inhibited hyperalgesia.

Insulin growth factor 1 receptor (IGF1R) has been identified as a potential RMT candidate based on observation that its ligand, IGF-1 is transported across the BBB. A panel of V_H_Hs, targeting the ectodomain of this receptor was isolated from a phage-displayed immune library [75]. Following humanization, the ability of several IGF1R-binding V_H_Hs to transmigrate in rat and human BBB models in vitro was confirmed [75,76]. When expressed in fusion with a murine antibody fragment, the resultant IGF1R-specific V_H_Hs were found to significantly increase brain and CSF exposure in mice and rats compared to the control [75]. IGF1R-targeting V_H_Hs conjugated to galanin—A systemically restricted neuroactive peptide that produces analgesia by binding GalR1 and GalR2 receptors expressed in the brain [77]—Induced a strong analgesic effect in a rat model of inflammatory hyperalgesia following a single dose injection [75], suggesting the ability of these IGF1R-binding V_H_Hs to act as delivery carriers across the BBB.

The above studies provided evidence for the feasibility of using sdAbs as carriers targeting a new generation of RMT receptors for the development of CNS therapeutics.

## 3. Single-Domain Antibodies as Treatments against Neurodegenerative Diseases

### 3.1. Protein-Misfolding Diseases (PMDs)

A vast majority of neurodegenerative diseases are associated with misfolded proteins that interact with each other to form large aggregates referred to as amyloid fibrils [78]. These complexes are insoluble, highly organized and extremely stable, and their accumulation is toxic to the cell. Although all PMDs share a common mechanism of action, the nature of the misfolded proteins differs between each disorder and dictates the identity of the disease. Alzheimer’s disease is caused by the accumulation of amyloid β (Aβ) peptides and Tau proteins, whereas the aggregation of α-synucleins (αSyn) is at the origin of Parkinson’s disease. Similarly, aggregates formed by huntingtin (Htt) proteins lead to Huntington’s disease while Prion disease is associated with the conversion of the normal, cellular prion protein (PrP^c^) into its disordered scrapie isoform (PrP^sc^), which accumulates into large oligomers. The formation of fibrils is a complex phenomenon involving several intermediate distinct structures [78,79]. The identification and characterization of the various species formed during the process is essential for the development of early diagnostic tools and new therapeutic strategies. This is, however, an extremely difficult task due to the insolubility and heterogeneity of the different forms involved in the process of fibril formation. This explains the lack of effective treatments against the devastating PMDs to date.

Single-domain antibodies represent a promising asset for the treatment of PMDs since they possess unique characteristics allowing them to access unprecedented epitopes (see Section 1). In addition, their high specificity and stability ensured the targeting of specific species under harsh solubilizing conditions along the process of fibril maturation. In this next section, we will review recent advances in the use of sdAbs for the diagnostic and treatment of the main PMDs affecting the CNS.

#### 3.1.1. Alzheimer’s Disease (AD)

The first description of AD goes back to 1906 [80]. However, it took several decades until it was finally established as a major neurodegenerative disorder. It is now considered the main cause of dementia accounting for up to 80% of all cases [81]. Patients affected with the disease suffer several symptoms including the loss of memory and cognitive functions. This is believed to be due to two main phenomena. First, the extracellular accumulation of Aβ peptides made of 39–42 amino acids to form amyloid plaques in the CNS [82] and second, the aggregation of hyperphosphorylated Tau proteins into Tau tangles inside neurons [83]. The presence of these aggregates or their precursor forms severely affects the normal function of neurons leading to cell death. In recent years, several efforts have been deployed to generate antibody fragments against Aβ and Tau aggregates in view of developing novel therapeutics for AD.

V_H_H B10 emerged from a synthetic phage-displayed library panned against biotinylated Aβ (1-40) fibrils [84]. This antibody was shown to bind specifically to mature amyloid fibrils as well as to protofibrils which were defined as the aggregated species forming prior to the assembly of more stable mature fibrils [85]. V_H_H did not interact with disaggregated peptides or other non-fibrillar Aβ oligomers. In addition, the authors demonstrated the antibody’s ability to stabilize protofibrils upon interacting with it, thus inhibiting mature fibril formation. However, B10 did not have the ability to disintegrate preformed fibrils. Similarly, another V_H_H isolated from a synthetic phage-displayed library, was shown to interact specifically with non-fibrillar Aβ (1-40) oligomers and prevent the formation of mature fibrils [86]. The antibody could not induce the disaggregation of already formed fibrils. In their report, the authors immobilized biotinylated Aβ (1-40) oligomers to select a conformation-specific binder that they named KW1. They demonstrated that the addition of KW1 to preformed Aβ oligomers prevented their synaptotoxic effect. Nevertheless, another report published two years later showed that Aβ oligomers formed in the presence of the same KW1 antibody were highly toxic [87], which seems to indicate a time-sensitive beneficial effect by the V_H_H. ni3A is a V_H_H that was isolated from a non-immune phage-displayed library using Aβ (1-42) as antigen [88]. This V_H_H bound to its target with high specificity and affinity and showed BBB-crossing abilities in vitro [89]. When tested in vivo [90], ni3A successfully detected Aβ deposits in a transgenic mouse model of AD, suggesting its potential as a diagnostic tool.

In contrast to the V_H_Hs described above, three additional ones were isolated from a phage-displayed library made from the blood of a llama immunized with a mixture of Aβ (1-42) monomers, small oligomers and fibrils [91]. These antibodies bound specifically to monomers and small oligomers formed exclusively by Aβ (1-42) but not to higher molecular-mass aggregates or fibrils or to Aβ (1-40)-originating species. One V_H_H in particular, V31-1, was found to inhibit the formation of amyloid fibrils and to prevent the toxic cellular effect of Aβ oligomers [91]. Another immunization campaign—this time using brain homogenates from an AD patient as immunogen in alpacas—Led to the identification of three V_H_Hs, PrioAD12, PrioAD13 and PrioAD120 targeting Aβ (1-40), Aβ (1-42) or Tau (1-16) peptides, respectively [92]. PrioAD12 had the ability to detect Aβ plaques in brain sections from an individual affected with AD while no detection (staining) was observed on sections from a normal brain. Finally, Aβ-specific V_H_s were isolated following immunization of a mouse with Aβ (1-42) peptides and construction of a phage-displayed V_H_ library [93]. Selected antibodies were found to interact with different regions of the full-length peptide and inhibit its cell toxicity. Moreover, one V_H_ (VH1.27), when tested for its ability to clear amyloid deposits in a mouse model of AD following intracranial injection was shown to significantly reduce the amyloid burden compared to the control.

Perchiacca and colleagues developed a new strategy to generate a series of sdAbs against disordered proteins [94,95,96,97]. They used defined algorithms to select motifs within disordered proteins that are predicted to participate in amyloid formation based on charge, hydrophobicity and propensity to form β-sheets [94]. They subsequently grafted peptides corresponding to the selected motifs into the CDR3 of a human V_H_ with good solubility characteristics. By using their technique, the authors generated a pool of antibodies against amyloidogenic epitopes within Aβ (1-42) peptides. The V_H_s demonstrated specific and sensitive recognition of Aβ monomers, soluble oligomers or fibrillar intermediates depending on the region covered by the grafted peptide and prevented toxicity induced by the targeted conformers [94]. It was later demonstrated that binding of the V_H_s with their amyloidogenic target led to the assembly of Aβ-V_H_s non-toxic complexes thereby preventing the formation of mature amyloid fibrils [95]. This technique was extended to construct one V_H_ specific for an aggregation-prone epitope within αSyn with the ability to inhibit fibrillization by the protein.

A similar grafting method was used to generate additional V_H_s targeting Aβ (1-42) or αSyn [98]. In this case, complementary peptides to the target sequence were designed based on interactions between amino acid sequences in the Protein Data Bank (PDB) and inserted into the CDR3 of a human V_H_. Resulting antibodies all showed specific binding to their respective target. In addition, one anti-αSyn V_H_ was tested for its neutralization potency in in vitro assays and demonstrated the ability to significantly reduce the aggregation of the targeted protein [98].

#### 3.1.2. Parkinson’s Disease (PD)

PD represents the second most common neurological disorder affecting approximately 10 million people worldwide, and this number is predicted to increase over the coming years due to population aging [99]. Hallmarks of the disease include the loss or degeneration of dopamine producing neurons leading to severe motor control impairment. At the molecular level, PD is associated with the appearance of intracellular fibrillar aggregates known as Lewy bodies (LB) or Lewy neurites composed mostly of αSyn [100]. These large inclusions are responsible for neuronal cell death. Therefore, antibodies targeting the small, αSyn protein represent a promising treatment against PD.

Three sdAbs recognizing αSyn have been described in addition to the ones mentioned in the previous section [94,101,102,103]. First, following immunization of a dromedary with monomeric αSyn and subsequent construction and screening of a phage-displayed library, a V_H_H (NbSyn2) interacting with the soluble form of the protein was identified [101]. Based on nuclear magnetic resonance (NMR) spectroscopy and X-ray crystallography, the epitope of NbSyn2 was mapped to the C-terminus of αSyn within the last four residues [104]. Interestingly, V_H_H also interacted with amyloid fibrils formed by αSyn suggesting that this region of the protein remains exposed following its aggregation. In this same report, the authors demonstrated that the binding of NbSyn2 to αSyn did not induce any structural changes nor did it have any effect on the kinetics of formation of fibrils [101]. However, the affinity of the binding decreased as the process of fibril formation progressed suggesting that there might be conformational rearrangements of the C-terminal region of αSyn upon fibril maturation.

The same group isolated a second V_H_H (NbSyn87) from a phage-displayed library generated from the blood of an immunized llama this time using a mutant of αSyn (A53T) [102], which has been associated with early onset of PD [105]. This antibody also interacted with a region encompassing the C-terminus of the monomeric αSyn distinct from the NbSyn2 epitope and had the ability to bind to amyloid fibrils without structural consequences. As was observed for NbSyn2, there was a time-dependent decrease in the affinity of the antibody for its amyloid target. Further characterization of NbSyn2 and NbSyn87 led to the observation that both V_H_Hs could inhibit the formation of mature fibrils in vitro [103]. They also had the ability to induce the conversion of αSyn from more stable oligomers into less stable oligomers significantly reducing the cellular toxicity caused by the protein.

The last αSyn-specific sdAb is a human V_H_ (VH14) against the non-amyloid component (NAC) region of monomeric αSyn, which was selected from a non-immune yeast-displayed scFv library [106]. Although it was shown to have the highest affinity for its target, this antibody failed to rescue the cytotoxicity induced by αSyn. Nevertheless, fusion of this domain antibody to a proteosomal targeting PEST motif increased its solubility and conferred the ability to induce αSyn clearance thereby reducing the toxic effect associated with protein aggregation both in situ [107] and in vivo [108]. When compared to NbSyn87-PEST, the VH14-PEST fusion demonstrated a more pronounced effect suggesting that the NAC region of αSyn is a preferable therapeutic target.

#### 3.1.3. Huntington’s Disease (HD)

HD is caused by an autosomal dominantly inherited CAG trinucleotide repeat expansion in the Htt gene [109]. Due to its high tendency to aggregate, the resulting mutant protein is at the origin of neuronal anomalies leading to cell death. In patients, this translates into numerous psychiatric and motor dysfunctions. There appears to be an inverse correlation between the length of expansion and age of onset. Although there are currently no curative treatments for HD, it certainly represents one of the most treatable neurological disorder since the molecular triggers are clearly defined. In this regard, sdAbs binding to mutant Htt have the potential to reduce its associated toxicity.

The use of a non-immune yeast-displayed scFv library led to the isolation of a human V_L_ sdAb targeting the first 20 amino acids of the Htt protein [110]. The V_L_ showed the same affinity for its target compared to its precursor scFv while achieving higher levels of cytoplasmic expression. However, inhibition of Htt aggregation demonstrated in a cell-free in vitro assay as well as in mammalian cells was only modest, requiring high amounts of the sdAb. In view of increasing the potency of V_L_, the same group submitted it to mutagenesis to remove its disulfide bond for efficient expression of natively folded sdAbs in the cytoplasm and subsequently increase its binding affinity [111]. The mutant, V_L_12.3, was able to strongly inhibit the formation of Htt aggregates and rescue cell toxicity in rat and yeast HD models. Adenoviral-delivery of this sdAb was shown to significantly improve behavior and neuropathology in a lentiviral mouse model of HD [112]. In contrast, when injected in transgenic HD mice, the antibody was found to increase the severity of the disease leading to a higher mortality rate. This was later attributed to a higher nuclear retention of Htt in the presence of V_L_12.3 in the transgenic HD mouse model [113].

Similarly, two more V_L_ domain antibodies (Happ1 and Happ3) targeting the proline-rich region of Htt were selected from a non-immune phage-displayed human scFv library [113]. Their capacity to reduce Htt-induced toxicity in cell culture increased compared to their scFv predecessors. Furthermore, they both had a greater ability to prevent neurodegeneration in a brain slice model of HD. Their mechanism of action involved an increased turnover rate of mutant Htt. Adenoviral-delivery of Happ1 demonstrated its efficacy in vivo in different mouse models of HD in which marked reduction of the disease-associated symptoms was observed following administration of the sdAb [112].

The first V_H_Hs (iV_H_H1–iV_H_H4) against the N-terminal region of Htt have been isolated from an immunized llama using phage display technologies [114]. Although the functionality of these sdAbs remains to be examined, they were found to interact with purified human wild-type and mutant Htt and also co-immunoprecipitated with both species following incubation with human HD brain lysates.

#### 3.1.4. Prion Diseases

Prion diseases, also known as transmissible spongiform encephalopathies (TSEs) comprise of a group of fatal transmissible neurodegenerative diseases caused by the misfolding of the cellular prion protein (PrP^c^) into the abnormally shaped scrapie prion protein (PrP^sc^). The emergence of the diseased state of the prion protein (PrP) can be spontaneous, genetic, or acquired [115]. In all cases, each newly formed PrP^sc^ acts as a template and promotes the conversion of more PrP^c^ leading to the assembly of large insoluble amyloid fibrils associated with neurotoxicity and spongiform change in the brain parenchyma. The most common TSE is the Creutzfeldt-Jakob disease with a new incidence rate of about 1–2 cases per million of population worldwide [116]. People suffering from this disorder show a wide variety of psychiatric symptoms that rapidly progress leading ultimately to death. Despite the severity of prion diseases, only two sdAbs targeting PrPs have been generated to date.

The first one, PrioV3 was isolated from a phage-displayed V_H_H library generated from the blood of dromedaries immunized with brain homogenates from scrapie-infected mice adsorbed on magnetic beads [117]. The V_H_H showed high affinity binding to a linear epitope at the C-terminus of both PrP^c^ and PrP^sc^. PrioV3 was shown to cross the BBB in vitro in rat and human brain endothelial cell lines via RMT [92,117]. Moreover, when injected intravenously in rats, the V_H_H was detected in the brain parenchyma suggesting its ability to cross the BBB in vivo. It also had the capacity to reduce PrP^c^ expression and PrP^sc^ accumulation in prion-permissive cells following its addition to the culture medium. When the treatment was prolonged over four days, PrP^sc^ was undetectable by Western blot suggesting complete and permanent inhibition of its replication by the antibody. Similar results were obtained in vivo in mice inoculated with scrapie-infected brain homogenates receiving a weekly dose of PrioV3 [92,117]. This treatment severely abrogated the accumulation of PrP^sc^ in the spleen of the animals. Finally, PrioV3 showed no sign of neurotoxicity in vitro.

Nb484 was selected from a pool of 14 V_H_Hs identified following llama immunization with murine PrPs and construction of phage-displayed V_H_H libraries [118]. This specific V_H_H showed the highest affinity for human PrPs. Assessment of its neutralizing properties revealed that the antibody could delay the formation of fibrils and abrogate the expression of PrP^sc^ in scrapie-infected murine cells. In addition, Nb484 was used as a crystallization chaperone, allowing the solution of the first crystal structures of the full length human PrP^c^ and a C-terminal truncated version of the protein, revealing novel structural insights on the early events of the conversion of PrP^c^ into PrP^sc^.

### 3.2. Glioblastoma Multiforme (GBM)

GBM is the most common type of brain tumors with the emergence of approximately 1000 new cases every year worldwide [119]. It is a highly aggressive malignancy showing rapid growth, intensive vascularization and predominant necrosis. Current treatments generally consist of maximal surgical resection followed by radiotherapy and chemotherapy. However, even with the use of these interventions, the prognosis remains extremely low and patients usually succumb to the disease within the first two years following diagnosis. This is in part due to the highly invasive nature of GBM and the difficulty of surgically removing all tumor cells. In addition, there is now growing evidence that the presence of chemotherapy and radiotherapy resistant stem-like cells within the tumor contributes to the resilience and recurrence of GBM [120]. Since early stages of the disease are mostly asymptomatic, current therapeutic strategies also suffer from late diagnosis. Alternative tools for the treatment and diagnosis of GBM are therefore urgently needed. Here we will review the different applications for sdAbs to improve current therapeutic modalities against GBM.

In view of identifying novel biomarkers for GBM, Jocevzka and colleagues prepared a phage-displayed V_H_H library from the blood of a llama immunized with a human GBM cell line enriched in stem-like cells [121,122]. Following several rounds of selection using protein extracts from diverse biological samples, three GBM-specific V_H_Hs were designated for further characterization. Identification of their antigen by mass spectrometry revealed two proteins, Trim28 and β-actin, which showed enrichment in GBM compared to control samples. The relevance of these proteins as GBM biomarkers remains to be determined. Using a similar approach, the same group isolated seven additional V_H_Hs specifically interacting with GBM antigens [123]. Initial Western blot and qPCR analyses complemented with bioinformatics demonstrated differential expression of some of the identified proteins in GBM compared to low grade gliomas suggesting their potential application as glioma class differentiation markers. Moreover, one antigen, mitochondrial translation elongation factor (TUFM) was isolated in a second independent screening by the same authors in which the selection of a V_H_H specific for GBM stem cells (GSCs) was achieved [124]. The specificity of the V_H_H for its target was confirmed by immunocytochemistry, and cytotoxicity assays demonstrated its profound effect on GSC growth.

VH-9.7 is a GSC-binding human V_H_ that emerged from a non-immune yeast-displayed human scFv library using a patient-derived GSC line for selection [125]. This sdAb showed selective binding to five GSC lines and successfully identified GSCs in mouse brain xenografts by flow cytometry. Its ability to detect and localize to GSCs was also demonstrated in vivo in mice harboring orthotopic GSC xenografts following intravenous injection of a fluorophore-conjugated VH-9.7.

The following study aimed to develop novel strategies to target GBM vasculature using an in vivo panning technique to isolate camelid phage-displayed sdAbs specifically accumulating in tumor vessels [126]. This led to the identification of the C-C7 V_H_H, which was later shown to target a distinct population of tumor vessels in mice xenografts as well as in GBM patient samples. The antibody also had the capacity to accumulate in the tumor vasculature following injection in mice harboring orthotopic xenografts while no antibodies were detected in normal brain vessels. Using a yeast-two-hybrid method, the antigen of C-C7 was identified as Dynactin-1-p150Glued, which was expressed exclusively on activated endothelial cells and may represent a valuable tumor vessel target. The antibody presented here could be used to assess the level of angiogenesis in GBM patients and determine the severity of the disease.

Finally, V_H_Hs targeting the epidermal growth factor receptor (EGFR) have been investigated as GBM therapeutic agents [127]. EGFR is well known to be overexpressed and mutated in a wide variety of tumors including GBM and has been extensively studied as an anti-cancer target [128,129]. V_H_Hs targeting this receptor were isolated from an immune phage-displayed library following llama immunization with overexpressing cell preparations [130]. These antibodies (ENb1 and ENb2) were selected specifically for their ability to prevent binding of the EGF ligand to the receptor via competitive elution strategies. When engineered for sustained on-site delivery by neural-stem cells, the V_H_Hs were shown to localize specifically in the tumor environment and inhibit EGFR signaling in vitro and to significantly reduce tumor growth in mouse models of malignant and invasive GBMs [127].

## 4. Single-Domain Antibodies as Neuroimaging Tools

### 4.1. Single-Domain Antibodies as Targeted Molecular Imaging Agents

Molecular imaging using advance and hybrid imaging modalities such as computed tomography (CT), positron emission tomography (PET), single photon emission computed tomography (SPECT), magnetic resonance imaging (MRI), and optical imaging, provide noninvasive means to characterize physiological processes and correlate molecular alterations with clinical outcomes. These technologies are improving early disease diagnosis, surgical guidance, patient stratification, and treatment monitoring [131]. Molecular imaging has advanced significantly during the last few decades through the identification of novel molecular targets and the development of multifunctional contrast agents along with new imaging instrumentation and analysis tools to extract quantitative data.

Targeted molecular imaging consists of an imaging probe linked to an agent that targets a specific biomarker of clinical relevance. Targeted molecular imaging agents have unique requirements that often differ from those of targeted therapeutic agents. In both cases, a high expression of the target antigen in the diseased versus normal tissue is required. However, for a targeted molecular imaging agent, a short half-life in circulation is preferable. Standard mAbs have a long half-life with slow liver clearance, which is a major hindrance for imaging applications, where a high contrast at early time points is critical for clinical applications [132]. The small size of sdAbs enables good tissue penetration and a fast clearance of the unbound fraction primarily via renal filtration (~60 kDa cutoff) [133,134,135]. This also allows the use of short-lived radionuclides, such as ^68^Ga (t_1/2_ = 68 min) or ^18^F (t_1/2_ = 109.8 min) for PET imaging which significantly reduces the patient’s exposure to radiation. A first-in human PET study using an anti-HER2 sdAb labeled with ^68^Ga-NOTA in female patients with metastatic breast cancer showed that imaging at 60–90 min provides suitable contrast to detect small and large tumor lesions with a fast blood clearance of the sdAb, such that only 7.2% of initial activity was remaining at 90 min [131].

The unique modularity of sdAbs to be engineered in different multivalent formats, including monomers, dimers, and pentamers provide additional flexibility to fulfill their antigen binding and pharmacokinetic characteristics to specific applications [136]. For instance, anti-EGFR sdAbs in monomer and pentamer formats showed to be particularly suitable for molecular optical imaging of glioblastoma tumors due to their respective short half-lives of 40 min and 80 min, while the same sdAbs engineered into a bivalent format fused with human IgG Fc have better potential to be exploited for therapeutic applications due to their extended half-lives (12.5 h) and enhanced avidity [136].

### 4.2. Single-Domain Antibodies for Imaging Brain Tumor Vasculature

The brain tumor vasculature represents a readily reachable target for molecular imaging due to its direct access via blood perfusion after intravenous administration. For brain tumors, such as GBM, assessment of tumor angiogenesis can provide information on the severity of the disease and guide appropriate treatment regimens [137,138]. Various tumor vascular targets that are overexpressed in the disease brain tissue and not in normal brain have been previously exploited by molecular targeted moieties for the non-invasive assessment of tumor angiogenesis using PET, optical imaging, and MRI. These include vascular endothelial growth factor receptor 2 (VEGFR2) [139], endothelial cell adhesion molecules (αvβ3 and αvβ5 integrins) [140], and insulin-like growth factor binding protein 7 (IGFBP7) [141,142]. IGFBP7, in particular, is a secreted protein that accumulates in the basement membrane of tumor endothelial cells, and its expression is believed to be associated with higher-grade gliomas [141,142,143]. Since the tumor’s malignancy is highly correlated with the degree of angiogenesis [137,138], the use of anti-IGFBP7 sdAb linked to a contrast agent for tumor vascular imaging could aid in the diagnosis and clinical management of brain tumors. In preclinical mouse models of GBM, an anti-IGFBP7 sdAb linked to a fluorophore was capable of non-invasively imaging the degree of angiogenesis [142]. Furthermore, bimodal optical-MRI contrast agents were developed by the bio-conjugation of anti-IGFBP7 sdAbs and the near-infrared fluorophore Cy5.5 to the surface of two types of nanoparticles, gadolinium-coated lipid particles for T1-weighted MRI imaging [144] and PEG functionalized-iron oxide nanoparticles for T2-weighted MRI imaging [145]. In both cases, after intravenous administration, the agents elicited an increased MRI contrast enhancement and fluorescent signal in a xenograft GBM tumor compared to a non-targeted nanoparticle. The molecular localization of the anti-IGFBP7 sdAb in the tumor brain vessels was further demonstrated by fluorescence microscopy [142].

### 4.3. Single-Domain Antibodies for Imaging Brain Targets

Due to the presence of the BBB, which limits the access of most biologics (i.e., proteins, peptides, antibodies) to the brain, radioligands used for PET imaging of CNS targets have been based on small molecular weight (<500 Da) molecules [146]. Targeted radioligands utilizing antibodies, antibody fragments, or sdAbs have been mainly developed for peripheral targets and used in different applications, including the detection of tumor markers, monitoring inflammatory processes, and visualization of antitumor immune responses [147]. However, a variety of strategies have been employed to allow the delivery of protein molecules across the BBB using both disruptive and non-disruptive methods.

As previously described, transmigration of antibodies across the BBB via RMT is a non-disruptive method for gaining access to brain targets (see Section 2). Using this strategy molecular imaging probes coupled to BBB carriers can be shuttled to the brain. For instance, taking advantage of the modularity of the BBB-transmigrating FC5 sdAb, a lipid-based nanoparticle was designed to encapsulate the anti-cancer drug doxorubicin and to display on its surface both a near-infrared (NIR) imaging agent and FC5 sdAb [148]. Upon intravenous injection in mice, in vivo optical imaging indicated increased brain delivery of the FC5-targeted versus non-targeted doxorubicin-containing liposomes. The optical fluorescent signal detected in vivo in the brain parenchyma correlated with the amount of doxorubicin delivered in the brain and measured ex vivo. Thus, this method allows for a non-invasive estimation of drug delivery into the brain.

BBB transmigration of macromolecules may also be achieved via adsorptive-mediated endocytosis through non-specific, charge-based interactions with the endothelial cell surface [149]. Endothelial cells are characterized by the presentation of negatively-charged clathrin-coated pits at the luminal surface, which can bind cationic proteins and facilitate their penetration through the BBB [150]. In an AD in vivo two-photon imaging study, sdAbs, selected for a basic isoelectric point (i.e., due to cationic amino acids) and for their binding to brain Aβ deposits or Tau inclusions were able to penetrate the BBB and bind to their respective brain target in vivo [135]. Interestingly, it was suggested that, in addition to the basic isoelectric pI, the molecular size of the sdAb was an important factor in the BBB penetration capability, as larger constructs (i.e., sdAb dimers) demonstrated reduced BBB penetration [151].

Some sdAbs have also been shown to interact with intracellular targets (i.e., penetrate cells) [152]. For instance, V_H_Hs against the astrocyte marker glial fibrillary acidic protein (GFAP) has been shown to cross the BBB, reach the brain tissue, and penetrate into astrocytes, as demonstrated by immunofluorescence studies on injected animal tissue sections [151]. This feature of sdAbs has the potential to open up “difficult to access” intracellular targets in the brain or within brain cell subtypes.

In summary, sdAbs hold promise for dynamic imaging compared to other antibody-based agents due to their small size that allows better tissue penetration, rapid and homogeneous tumor/brain accumulation and fast blood clearance, which results in high tissue-to-background noise ratios. Single-domain antibodies are versatile, stable in very harsh conditions (pH, temperature), easy to conjugate to different imaging probes, and relatively safe due to their high specificity.

## 5. Conclusions

Singe-domain antibody technologies are ‘coming of age’ with many being tested in clinical trials. Several notable advantages of this compact antibody format, including ease of engineering, stability, recognition of unusual epitopes, and versatility for creating bi- and multifunctional molecules, have resulted in sdAbs being poised to address some of the most difficult target and disease spaces, most notably those of the CNS. CNS diseases are among the most difficult to treat not only because therapeutic targets (e.g., misfolded proteins, ion channels and G-protein coupled receptors) are very complex, but also because they are ‘hidden’ behind brain barriers and are thus difficult to access systemically. Selectivity of targeting of receptor/channel subtypes, often in specific activation states, specific targeting of point mutations, or epitopes ‘embedded’ in misfolded proteins present unique challenges, often difficult to address by either synthetic molecules or mAbs. While ‘precision’ targeting of desired epitopes is achievable by both sdAbs and mAbs, compact sdAb format could improve access to hidden epitopes. One distinct advantage of this format is improved diffusion in brain tissue after direct intracerebral administration, and enhanced brain tissue penetration after intrathecal infusion via perivascular flow. Furthermore, sdAbs are proving to be a versatile format for designing BBB carriers that could be easily combined in various display linkages (mono-, bi-, multivalent) with therapeutic monoclonal antibodies and other therapeutic cargos (peptides, proteins, nanocarriers, and imaging agents). The pipeline of sdAbs, both camelid and human, raised against CNS targets from naïve or immune libraries and tested in preclinical models is growing with prospects for entry into clinical testing in the near future. With parallel and significant progress in the development of BBB-delivery technologies based on sdAbs, the field of CNS, so far dominated by small molecule therapeutics, is slowly but steadily progressing into a new era of biological treatments, most notably antibody therapies for chronic neurodegenerative diseases.

## Figures and Tables

**Table 1:** Overview of single-domain antibodies developed for central nervous system applications.

Product Name	Target	sdAb Type	Source	References
**BBB Shuttles**				
TBX4	TfR1	V_NAR_	Synthetic phage-displayed V_NAR_ library	[66]
IGF1R-3	IGF1R	V_H_H	Immune phage-displayed V_H_H library	[75,76]
FC5	TMEM-30A	V_H_H	Non-immune phage-displayed V_H_H library	[20,70,71,72,73,74]
FC44	Unknown	V_H_H	Non-immune phage-displayed V_H_H library	[20,70,71]
**Neurodegenerative diseases**				
**Alzheimer’s disease**				
B10	Mature Aβ (1-40) fribrils and protofibrils	V_H_H	Synthetic phage-displayed V_H_H library	[84]
KW1	Non-fibrillar Aβ (1-40) oligomers	V_H_H	Synthetic phage-displayed V_H_H library	[86,87]
ni3A	Aβ (1-42) deposits	V_H_H	Non-immune phage-displayed V_H_H library	[88,89,90]
V31-1	Monomers and small Aβ (1-42) oligomers	V_H_H	Immune phage-displayed V_H_H library	[91]
PrioAD12	Aβ (1-40) peptide	V_H_H	Immune phage-displayed V_H_H library	[92]
PrioAD13	Aβ (1-42) peptide	V_H_H	Immune phage-displayed V_H_H library	[92]
PrioAD120	Tau (1-16) peptide	V_H_H	Immune phage-displayed V_H_H library	[92]
VH1.27, VH1.28, VH2.8	Aβ (1-42) peptide	V_H_	Immune phage-displayed mouse V_H_ library	[93]
Aβ (1-10), Aβ (3-12), Aβ (6-15), Aβ (9-18), Aβ (12-21), Aβ (15-24), Aβ (18-27), Aβ (21-30), Aβ (24-33), Aβ (27-36), Aβ (30-39), Aβ (33-42)	AB monomers, soluble oligomers or fibrils	V_H_	Grafted amyloid-motif antibodies (Gammabody)	[94,95,96,97]
DesAb-Aβ	Aβ (15-21) peptide	V_H_	Gammabody	[98]
**Parkinson’s disease**				
αSyn (69-78)	αSyn fibrils	V_H_	Gammabody	[95]
DesAb-D, DesAb-E, DesAb-F	αSyn (61-67) or αSyn (70-76) peptide	V_H_	Gammabody	[98]
NbSyn2	Monomeric αSyn and mature fibrils	V_H_H	Immune phage-displayed V_H_H library	[101,102,104]
NbSyn87	Monomeric αSyn(A53T) and mature fibrils	V_H_H	Immune phage-displayed V_H_H library	[102,103,107,108]
VH14	Monomeric αSyn	V_H_	Non-immune yeast-displayed human scFv library	[106,107,108]
**Huntington’s disease**				
V_L_12.3	Htt protein	V_L_	Non-immune yeast-displayed human scFv library	[110,111,112,113]
Happ1, Happ3	Htt protein	V_L_	Non-immune phage-displayed human scFv library	[112,113]
iV_H_H1, iV_H_H2, iV_H_H3, iV_H_H4	Htt protein	V_H_H	Immune phage-displayed V_H_H library	[114]
**Prion diseases**				
PrioV3	PrP^c^ and PrP^sc^	V_H_H	Immune phage-displayed V_H_H library	[92,117]
Nb484	MoPrP (23-230)	V_H_H	Immune phage-displayed V_H_H library	[118]
**Glioblastoma multiforme**				
Nb237	TRIM28	V_H_H	Immune phage-displayed V_H_H library	[122]
Nb141	β-actin	V_H_H	Immune phage-displayed V_H_H library	[122]
Nb10	ACTB/NUCL complex	V_H_H	Immune phage-displayed V_H_H library	[123]
Nb79	VIM	V_H_H	Immune phage-displayed V_H_H library	[123]
Nb179	NAP1L1	V_H_H	Immune phage-displayed V_H_H library	[123]
Nb225	TUFM	V_H_H	Immune phage-displayed V_H_H library	[123]
Nb314	DPYSL2 and MTHFD1	V_H_H	Immune phage-displayed V_H_H library	[123]
Nb394	CRMP1	V_H_H	Immune phage-displayed V_H_H library	[123]
Nb395	ALYREF	V_H_H	Immune phage-displayed V_H_H library	[123]
Nb206	TUFM	V_H_H	Immune phage-displayed V_H_H library	[124]
VH-9.7	GSC	V_H_	Non-immune yeast-displayed human scFv library	[125]
C-C7	Dynactin-1-p150Glued	V_H_H	Non-immune phage-displayed V_H_H library	[126]
ENb1, ENb2	EGFR	V_H_H	Immune phage-displayed V_H_H library	[127,130]
**Neuroimaging**				
EG(2)	EGFR	V_H_H	Immune phage-displayed V_H_H library	[136]
sdAb 4.43	IGFBP7	V_H_H	Immune phage-displayed V_H_H library	[142,144,145]
FC5	TMEM-30A	V_H_H	Non-immune phage-displayed V_H_H library	[148]
R3VQ	Aβ (1-42) peptide	V_H_H	Immune phage-displayed V_H_H library	[135]
A2	Phospho-Tau protein	V_H_H	Immune phage-displayed V_H_H library	[135]
mVHH A10, mVHH E9, mVHH E3	GFAP	V_H_H	Immune ribosome-displayed V_H_H library	[151]
